# Optimizing nitrogen fertilizer amount for best performance and highest economic return of winter wheat under limited irrigation conditions

**DOI:** 10.1371/journal.pone.0260379

**Published:** 2021-11-29

**Authors:** Pin Zhang, Yi-kang Qi, Hong-guang Wang, Jian-ning He, Rui-qi Li, Wei-li Liang

**Affiliations:** 1 College of Agronomy, Hebei Agricultural University, Baoding, Hebei, China; 2 State Key Laboratory of North China Crop Improvement and Regulation, Baoding, Hebei, China; 3 Key Laboratory of Crop Growth Regulation of Hebei Province, Baoding, Hebei, China; Soil and Water Resources Institute ELGO-DIMITRA, GREECE

## Abstract

Inappropriate water and fertilizer management can lead to unstable crop yields. Excessive fertilization can potentially cause soil degradation and nitrogen (N) leaching. The aim of this study was to explore the optimal N application rate on two wheat varieties with different nitrogen responding under limited water irrigation at three experimental sites in the Piedmont plain of the Taihang Mountains, China. A two-year field experiment was conducted to explore the effects of five N application rates (N0, N120, N180, N240, and N300) on winter wheat growth, leaf area index, aboveground biomass, grain yield, grain N accumulation, and net return. The results showed that N application rate significantly affected leaf area index, aboveground biomass, grain yield, and harvest index. Variety and variety × N rate interactions had a significant effect on few indicators. Compared with N0, N180 improved leaf area index, aboveground biomass, grain yield, and grain N accumulation. Compared with N240 and N300, N180 increased the harvest index and N harvest index, without significantly reducing grain yield or grain N accumulation, while enhancing a higher N use efficiency. Fertilizers applied in the ranges of 144.7–212.9 and 150.3–247.0 kg ha^-1^ resulted in the highest net return for the KN199 and JM585 varieties, respectively. Our study provides a sound theoretical basis for high-efficiency fertilizer utilization in sustainable winter wheat production in the Piedmont plains of the Taihang Mountains of China.

## Introduction

Wheat (*Triticum aestivum L*.) plays a highly significant role in the nutrition of the Chinese population [1]. Further, the North China Plain (NCP) is one of the primary crop production areas in China, where typical winter wheat is planted in approximately 61% of the total arable land [2]. Winter wheat production in this area plays an important role in ensuring food security and avoiding energy crises in China [3]. However, to continuously maximize crop yield, farmers frequently use nitrogen fertilizers and irrigation excessively. Meanwhile, by and large, groundwater irrigation is the primary irrigation source used across farmlands in the region. Thus, as a result of the excessive exploitation of groundwater, decreasing water availability for agricultural production has gradually restricted the sustainable development of local agriculture [4–6]. Concomitantly, the excessive use of nitrogen fertilizers threatens the ecological safety of the area [7,8] and may increase greenhouse gas emissions [9]. Therefore, increasing winter wheat yield while reducing water and fertilizer inputs, maintaining sustainable crop production, and improving the agroecological environment have all been imperative to achieve the sustainable intensification of agricultural systems in the North China Plain [10].

Sustainable agricultural intensification needs to optimize genotype × environment × management interactions for each target environment [11]. Over the past decades, the development of wheat varieties has contributed greatly to yield increases [12]. Nitrogen absorption by winter wheat is reported significantly correlated with different efficiency varieties [13]. Thirty-nine superior wheat varieties were planted at five N fertilization levels ranging from 0 to 350 kg ha^-1^. The results showed that all interactions between variety and N-rate were highly significant for the total N absorption and N use efficiency [14]. Total N absorption by high-N efficiency cultivars was higher than that of low-N efficiency cultivars [15]. In the NCP, the range of N input is 117–455 kg ha^-1^, but only 35% of the applied N fertilizer is absorbed by winter wheat [16,17], which leads to massive N loss and subsequent negative environmental impacts [18]. Selecting high-efficiency wheat varieties and optimizing agronomic management practices to increase water/nitrogen use efficiency (NUE) is an effective strategy to increase crop yield and reduce associated environmental costs [19]. The most suitable N application rate varies with planting area and wheat variety. Mehrabi et al. [20] showed that there was no significant difference between 150 and 300 kg N ha^-1^ in Shiraz, Iran. The optimal N application rate is 150 kg N ha^-1^. In turn, Xin et al. [12] showed that 150–210 kg N ha^-1^ in winter wheat cultivated in the NCP resulted in higher productivity, higher NUE and lower N_2_O emissions.

Although previous studies have optimized fertilizer input to maximize crop yields or net return, few studies have conducted multiple experiments in the Piedmont plains of the Taihang Mountains to investigate the effects of varying N application rates on wheat varieties with differences in factors affecting sustainable agriculture, including N efficiency, wheat yield, N uptake, and net return. Thus, understanding and mastering the N absorption-related in wheat varieties under different N application rates and limited water availability can aid in the development of a reasonable fertilization system to promote sustainable agricultural development, further maintaining crop yield and N absorption, and reducing soil erosion hazards. In this study, a two-year field experiment was conducted on winter wheat at three locations to determine the effects of the use of varieties and N application rates on wheat growth, grain yield, grain N accumulation, and net return. Additionally, the appropriate winter wheat varieties and N fertilization ranges to simultaneously optimize the grain yield, grain N accumulation, and net return were determined. The results of this study provide a technical reference for the production of high winter-wheat yields while promoting the synergistic utilization of varieties and nutrients in similar plains around the world.

## Materials and methods

### Experimental site description

**Fig 1:**
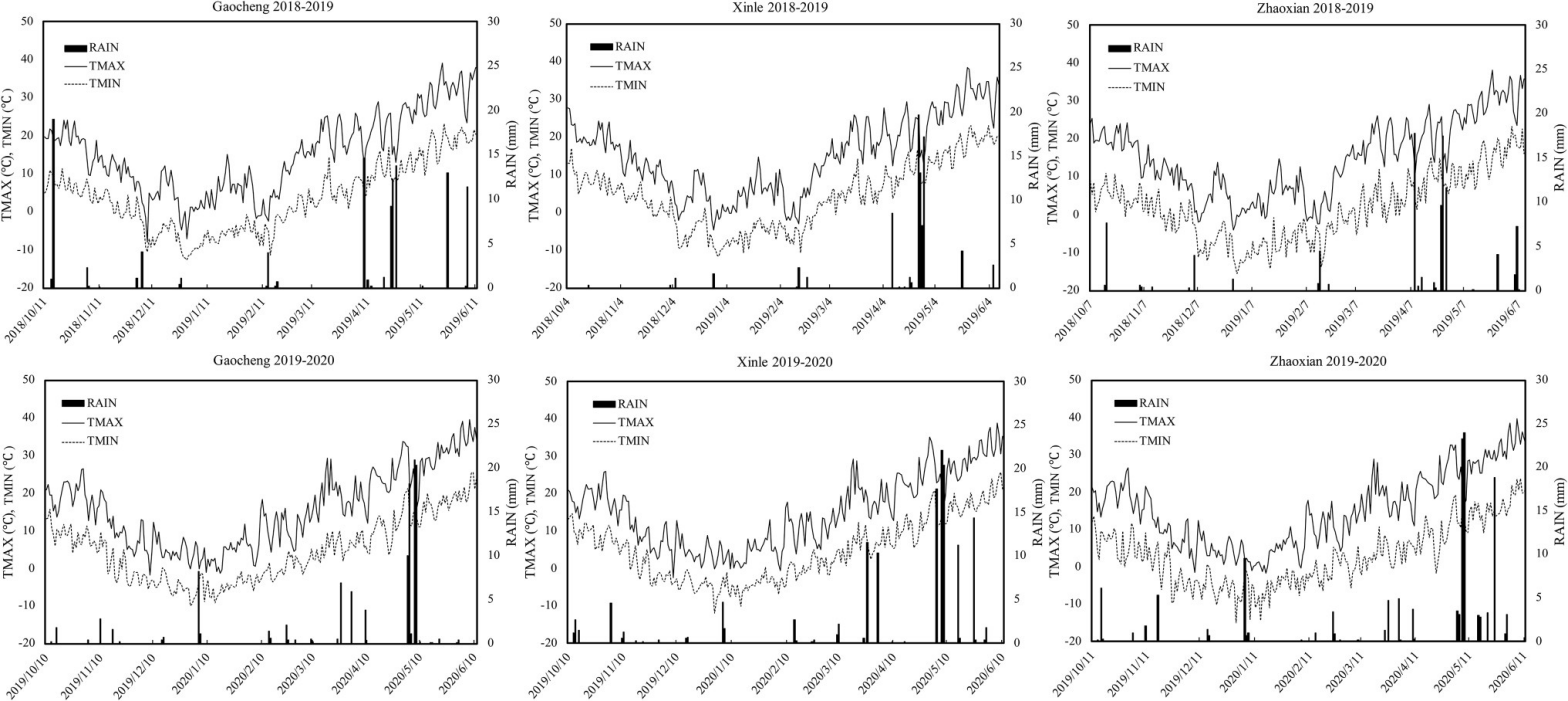
Daily rainfall (RAIN), maximum temperature (TMAX) and minimum temperature (TMIN) in three experimental sites during 2018–2020 seasons.

A field experiment was conducted in three representative locations in the Piedmont plain during the 2018–2019 and 2019–2020 winter wheat growing seasons in Gaocheng Liujiazhuang Village (37°96’ N, 114°89’ E), Xinle Zhongtong Village (38°40’ N, 114°71’ E), and Zhaoxian Northern Agricultural Park (38°40’ N, 114°71’ E). The study site in Gaocheng has a temperate, semi-humid, and continental monsoon climate, with an annual average temperature of 12.5°C and an average annual precipitation of 494.0 mm. On the other hand, the study site in Xinle is located at the eastern foot of the Taihang Mountains, on the sloped plain in front of the mountains. The site has a warm temperate, semi-humid monsoon continental climate, with an average temperature of 12.3°C and an average annual precipitation of 428.9 mm. In turn, the study site in Zhaoxian is located on the Piedmont alluvial plain in the middle section of the eastern foothills of the Taihang Mountains. This site has a warm temperate, semi-humid monsoon continental climate with an annual average temperature of 13°C and an average annual precipitation of 502.5 mm. The total amounts of precipitation during the wheat growing season in 2018–2019 and 2019–2020 were 94.4 mm and 135.2 mm at Zhaoxian, 111.4 mm and 112.0 mm at Gaocheng, and 81.3 mm and 139.4 mm at Xinle, respectively. Monthly total precipitations and mean temperatures are shown in Fig 1. The main characteristics of the 0–20 cm and 0–40 cm soil layers in the experimental region at the beginning of the experiment are shown in Table 1.

**Table 1:** The status of top soil before seeding in 2018 and 2019.

Growing season	Soil layer(cm)	Experiment Location	Soil bulk density (g cm^-3^)	Organic matter (g kg^-1^)	Available N (mg kg^-1^)	Available P (mg kg^-1^)	Available K (mg kg^-1^)
2018–2019	0–20	Gaocheng	1.64	21.30	146.42	22.50	169.60
		Xinle	1.78	19.58	118.42	10.40	108.20
		Zhaoxian	1.66	20.42	135.92	22.10	152.30
	20–40	Gaocheng	1.67	5.33	60.00	5.20	146.80
		Xinle	1.76	4.89	56.58	1.21	91.70
		Zhaoxian	1.73	5.02	53.08	2.70	111.20
2019–2020	0–20	Gaocheng	1.65	20.73	140.75	22.59	162.92
		Xinle	1.72	17.58	118.73	10.91	112.95
		Zhaoxian	1.68	18.98	133.43	21.53	149.99
	20–40	Gaocheng	1.69	5.05	56.70	5.33	147.76
		Xinle	1.75	4.44	53.57	1.42	94.73
		Zhaoxian	1.74	4.87	51.58	2.61	114.26

### Experimental design

The experiment adopted a two-factor split plot design at three experimental locations. The main plots were arranged for varieties KN199 (low nitrogen tolerant and high yield) and JM585 (nitrogen sensitive) previously studied [21]. The subplots were arranged for N application with five N fertilizer rates, namely, 0, 120, 180, 240, and 300 kg N ha^-1^ (referred to as N0, N120, N180, N240, and N300, respectively). Main plots were 50 m long ×10 m wide and subplots were 9 m long ×5 m wide in size, with a 1.0-m buffer zone between plots to minimize the effects of adjacent plots. In sum, there were 30 treatments with three replicates each. Both wheat varieties were sown at a rate of 180 kg seed ha^-1^. At the third-leaf growth stage, seedlings are thinned to attain a plant population density of 3.3 million basic seedlings per hectare. In the 2018–2019 winter growing season, plots in Gaocheng, Xinle, and Zhaoxian were planted on October 11, 4, and 7, respectively, and the crops were harvested on June 11, 4, and 7, respectively. Meanwhile, in the 2019–2020 growing season, plots in Gaocheng, Xinle, and Zhaoxian were planted on October 10, 10 and 11, respectively, and the crops were harvested on June 15, 10 and 11, respectively. This experiment was carried out under limited water conditions: during the whole growing season, only 60 mm water were applied at the jointing stage. N fertilizer was spread before irrigation at jointing stage, a water meter was connected at the water outlet of the pump, then the water pipe was connected to the plots in order to strictly control water quantity at 60 mm.

Nitrogen, phosphorus and potassium fertilizers were applied using urea (46.7% N), calcium superphosphate (16% P_2_O_5_), and potassium sulfate (60% K_2_O), respectively. The base fertilization before seeding was applied by spreading the fertilizer on the surface and mixing it into the soil with a rotary cultivator. P and K fertilizers were applied at 120 kg ha^-1^ and 150 kg ha^-1^, respectively, as base fertilizers; in turn, N fertilizer was split in two portions applied as base fertilizer (50%) and topdressing (50%) at the jointing stage. Herbicides (spray 15% Tribenuron 10 g hm^-2^) were applied before sowing and pesticides (mix 10% imidacloprid with 50 kg of water and spray) were applied at flowering period, so that the plots were kept free of weeds, insects, and diseases during the growing seasons.

### Measurements and calculations

#### Leaf area index (LAI)

At anthesis, the leaf area of 20 representative plants from each plot was measured using a LI-3000C portable leaf area meter (LI-COR, Lincoln Nebraska, USA).

#### Aboveground biomass

At maturity, 20 representative plants were selected from each plot. Each plant was divided into grain, stem and sheath, leaves, and the spike axis and glumes, and oven-dried at 105°C for 30 min and subsequently dried at 75°C to constant weight. The plant mass was determined using an electronic balance. The total accumulation of dry matter of each organ per hectare was calculated by sampling the number of plants and the basic seedling number per hectare.

#### Grain yield and harvest index

At grain maturity, an area of 2 m^2^ of undisturbed wheat was randomly selected to measure yield. Grains were air-dried and weighed after harvest, samples were taken and oven-dried for measuring moisture content. Yields were normalized at 13% moisture content.


$$\text{Harvest}\;\text{index}\;(\text{HI})=\frac{\text{Grain}\;\text{yield}\;\left(\text{kg}\;{\text{ha}}^{-1}\right)}{\text{Aboveground}\;\text{biomass}\;\left(\text{kg}\;{\text{ha}}^{-1}\right)}$$ (1)

#### N accumulation, N harvest index and N use efficiency

The various organs of the wheat plants whose dry matter was measured at maturity were ground to determine N accumulation. Dried samples were ground, extracted with H_2_SO_4_-H_2_O_2_, and analyzed for total N content (%) using a continuous-flow auto-analyzer (AutoAnalyzer 3; Bran + Luebbe, Noderstedt, Germany) [22].

Total N accumulation, N harvest index (NHI) and NUE were calculated according to the method described by Zhang et al. [23].


$$\text{Total}\;\text{N}\;\text{accumulation}\;\left({\text{kg}\;\text{ha}}^{-1}\right)=\text{N}\;\text{content}\;\left(\text{\%}\right)\;\times \;\text{aboveground}\;\text{biomass}\;\left({\text{kg}\;\text{ha}}^{-1}\right)$$ (2)

$$\text{NHI}=\frac{\text{Grain}\;\text{N}\;\text{accumulation}\;\left({\text{kg}\;\text{ha}}^{-1}\right)}{\text{Total}\;\text{N}\;\text{accumulation}\;\left({\text{kg}\;\text{ha}}^{-1}\right)}$$ (3)

$$\text{NUE}=\frac{\text{Grain}\;\text{yield}\;\left({\text{kg}\;\text{ha}}^{-1}\right)}{\text{Total}\;\text{N}\;\text{accumulation}\left({\text{kg}\;\text{ha}}^{-1}\right)}$$ (4)

#### Net return

Net return was calculated as follows [24]:

Nr=Gp−Ic−Fc−O (5)

where, Nr is the net return (CNY ha^-1^), Gp is the gross profit (CNY ha^-1^), Ic is the irrigation cost (CNY ha^-1^), Fc is the fertilizer cost (CNY ha^-1^), and O represents other costs (CNY ha^-1^).

### Data analysis

Analysis of variance (ANOVA) was performed using IBM SPSS Statistics for Windows, version 25 (IBM Corp., Armonk, NY, USA). Variety and fertilization rate were used as the primary effects and two-way interactions were included as well. All treatment means were compared for significant differences using Duncan’s multiple range test at a significance level of *P* = 0.05 [25].

## Results

### Leaf area index, aboveground biomass, grain yield, and harvest index

**Fig 2:**
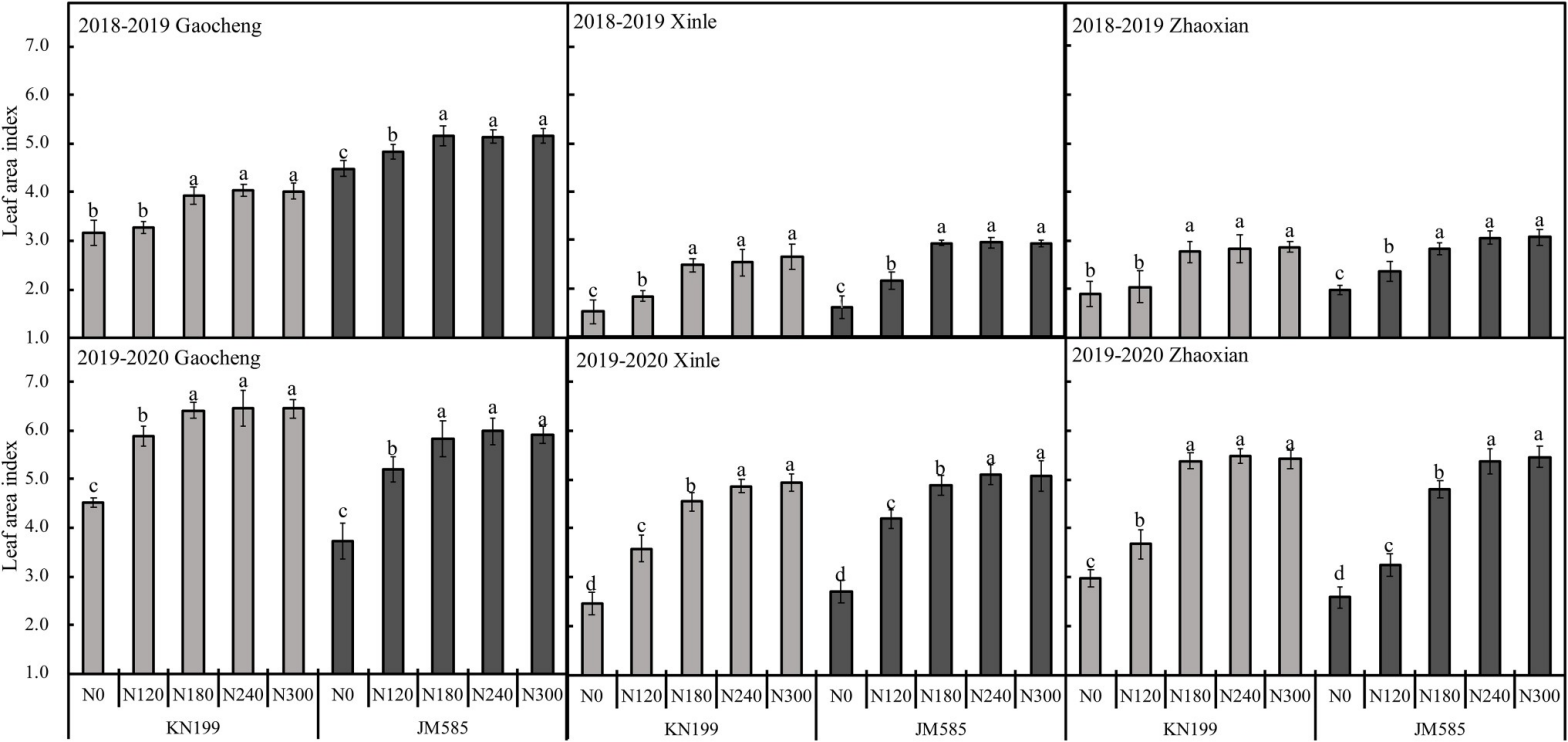
Leaf area index at anthesis of different winter wheat varieties under different nitrogen fertilization rates in three experimental sites during 2018–2020 seasons. Error bars represent one standard deviation from the mean. Letters above the bars are comparison results of leaf area index between different nitrogen treatments of the same variety. There is no significant difference between treatments with same letters.

Location, variety and N rate interaction significantly affected LAI in 2018–2020. The interaction of L×V significantly affected the LAI in 2018–2020, and L×V only affected the LAI in 2019–2020 (Table 2). As shown in Fig 2, LAI recorded for KN199 under the N180 treatment increased significantly by 24.11%, 63.10%, and 45.79% at Gaocheng, Xinle, and Zhaoxian, respectively, compared with the N0’treatment in 2018–2019 growing season. In 2018–2019, LAI for JM585 under the N180 treatment increased significantly by 15.08%, 82.89%, and 42.11% at Gaocheng, Xinle, and Zhaoxian, respectively, compared with the N0’treatment. Moreover, in 2019–2020, at Xinle and Zhaoxian experimental locations, the LAI of JM585 increased significantly by 4.34% and 11.95%, respectively when the N input increased from N180 to N240.

**Table 2:** Significance level (*P* values) of the effects of location, variety and N rate on leaf area index, aboveground biomass, grain yield, HI, total N accumulation, grain N accumulation, NHI and NUE of winter wheat.

Growing season	Treatment	Leaf area index	Aboveground biomass	Grain yield	HI	Total N accumulation	Grain N accumulation	NHI	NUE
2018–2019	Location (L)	<0.001	<0.001	<0.001	<0.001	<0.001	<0.001	<0.001	<0.001
	Variety (V)	<0.001	<0.001	0.021	<0.001	0.547	<0.001	<0.001	0.017
	N rate (N)	<0.001	<0.001	<0.001	<0.001	<0.001	<0.001	<0.001	<0.001
	L×V	<0.001	<0.001	0.536	<0.001	<0.001	<0.001	<0.001	0.005
	L×N	0.983	<0.001	0.090	<0.001	<0.001	<0.001	<0.001	<0.001
	V×N	0.365	0.001	0.845	0.196	<0.001	<0.001	0.099	<0.001
	L×V×N	0.433	0.003	0.999	0.021	0.128	0.178	0.013	<0.001
2019–2020	Location (L)	<0.001	<0.001	<0.001	0.170	<0.001	<0.001	<0.001	<0.001
	Variety (V)	<0.001	0.004	<0.001	<0.001	<0.001	<0.001	<0.001	<0.001
	N rate (N)	<0.001	<0.001	<0.001	<0.001	<0.001	<0.001	<0.001	<0.001
	L×V	<0.001	0.005	<0.001	<0.001	0.557	0.005	<0.001	<0.001
	L×N	<0.001	0.002	0.042	0.093	<0.001	<0.001	<0.001	<0.001
	V×N	0.820	0.017	0.426	0.170	0.132	0.302	0.832	0.018
	L×V×N	0.199	0.003	0.101	0.849	0.003	0.003	0.021	0.471
2018–2020	Growing season (S)	<0.001	<0.001	<0.001	<0.001	<0.001	<0.001	<0.001	<0.001
	Location (L)	<0.001	<0.001	<0.001	<0.001	<0.001	<0.001	<0.001	<0.001
	Variety (V)	<0.001	<0.001	<0.001	<0.001	<0.001	<0.001	<0.001	<0.001
	N rate (N)	<0.001	<0.001	<0.001	<0.001	<0.001	<0.001	<0.001	<0.001
	S×L	<0.001	<0.001	<0.001	0.001	<0.001	<0.001	<0.001	<0.001
	S×V	<0.001	<0.001	<0.001	<0.001	<0.001	<0.001	<0.001	<0.001
	S×N	<0.001	<0.001	<0.001	0.005	<0.001	0.216	<0.001	0.019
	L×V	<0.001	<0.001	<0.001	<0.001	0.001	<0.001	<0.001	<0.001
	L×N	<0.001	<0.001	0.002	0.004	<0.001	<0.001	<0.001	<0.001
	V×N	0.378	<0.001	0.313	0.023	<0.001	<0.001	0.911	0.036
	S×L×V	<0.001	0.143	<0.001	<0.001	0.109	0.264	0.013	<0.001
	S×L×N	<0.001	<0.001	0.672	0.007	<0.001	<0.001	<0.001	0.197
	S×V×N	0.686	0.856	0.961	0.871	0.033	0.078	0.110	<0.001
	L×V×N	0.466	0.053	0.399	0.352	0.005	0.009	0.017	<0.001
	S×L×V×N	0.265	<0.001	0.522	0.188	0.020	0.013	0.013	<0.001

Note: HI: Harvest index; NHI: Nitrogen Harvest index; NUE: Nitrogen use efficiency.

**Fig 3:**
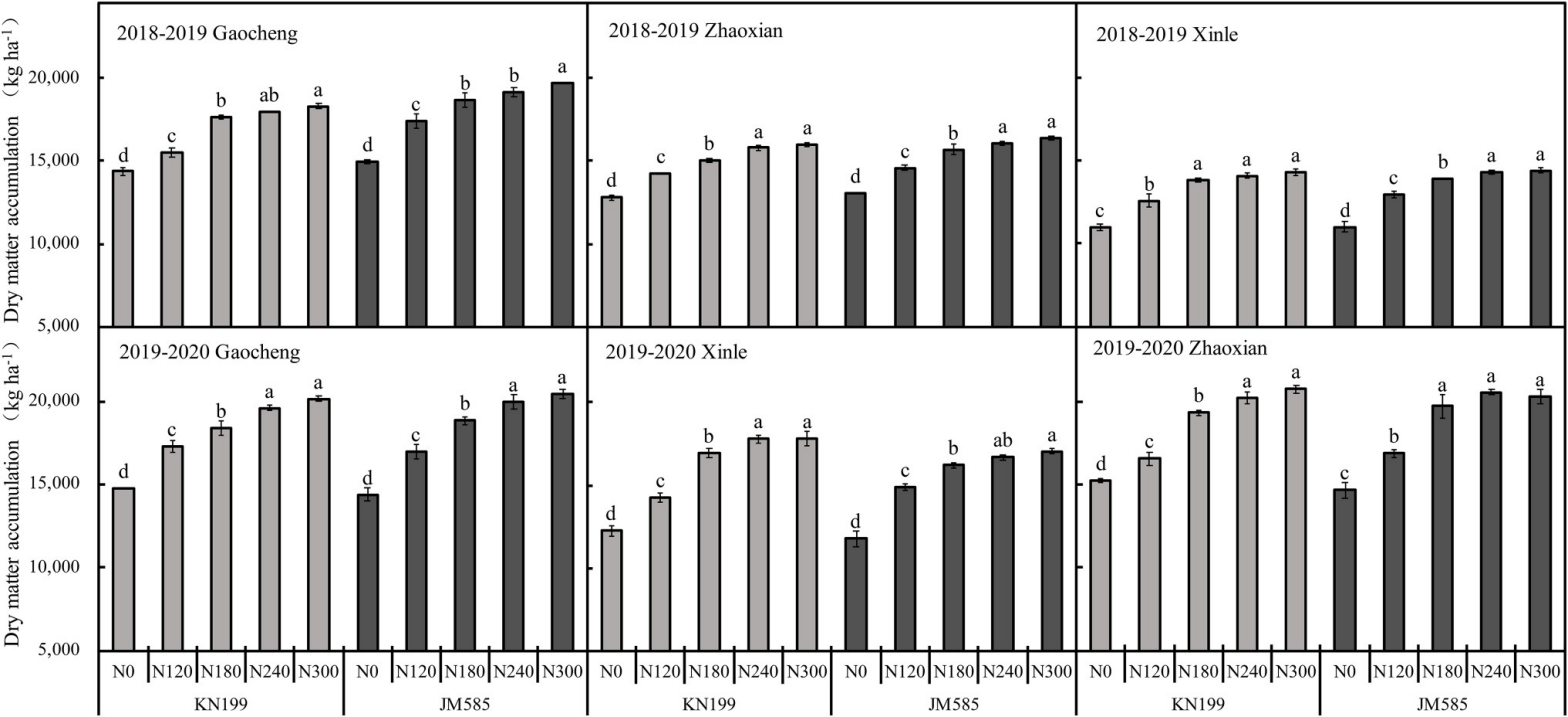
Aboveground biomass at maturity of different winter wheat varieties under different nitrogen fertilization rates in three experimental sites during 2018–2020 seasons. Error bars represent one standard deviation from the mean. Letters above the bars are comparison results of leaf area index between different nitrogen treatments of the same variety. There is no significant difference between treatments with same letters.

Location, variety, and N rate significantly affected aboveground biomass in the two growing seasons considered herein. The interaction of L×V, L×N and V×N significantly affected the aboveground biomass in 2018–2020 (Table 2). As shown in Fig 3, the aboveground biomass of KN199 under the N180 treatment increased significantly by 23.12%, 25.60%, and 17.66% at Gaocheng, Xinle, and Zhaoxian, respectively, compared with the N0’treatment in 2018–2019 growing season. In the 2018–2019 growing season, the aboveground biomass of JM585 increased significantly by 24.69%, 26.56%, and 20.37% at Gaocheng, Xinle, and Zhaoxian, respectively, under the N180 treatment, compared to the N0’treatment. Furthermore, when N input increased to N240 and N300, aboveground biomass significantly increased in Xinle and Zhaoxian experimental locations; However, grain yield did not increase significantly, while dry matter accumulated in vegetative organs was the portion of the plant body whose dry matter increased more. In the 2019–2020 growing season, grain yield and dry matter for the two winter wheat varieties were consistent with those observed in the previous season.

**Fig 4:**
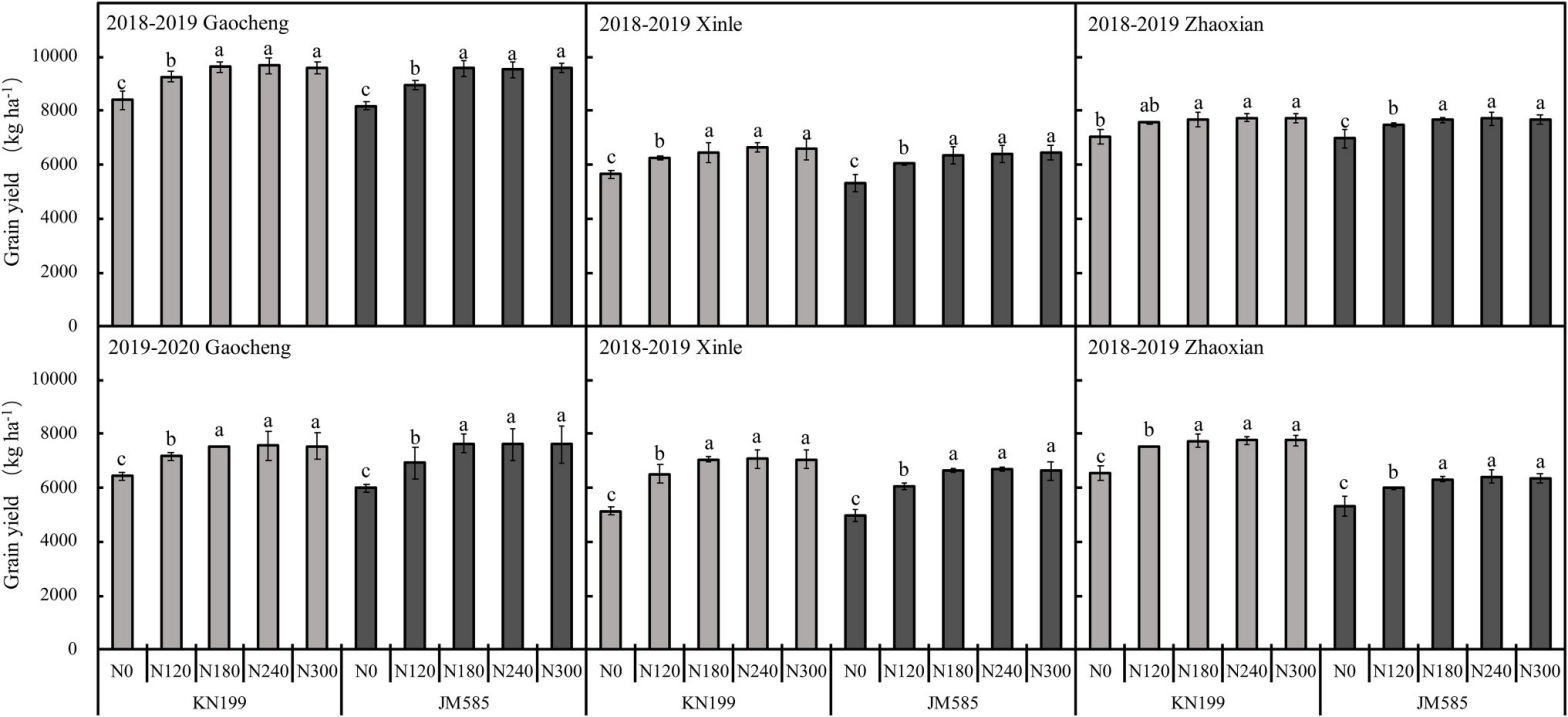
Grain yield of different winter wheat varieties under different nitrogen fertilization rates in three experimental sites during 2018–2020 seasons. Error bars represent one standard deviation from the mean. Letters above the bars are comparison results of leaf area index between different nitrogen treatments of the same variety. There is no significant difference between treatments with same letters.

Location, variety, and N rate significantly affected grain yield in the two growing seasons considered herein. The interaction of L×V significantly affected the grain yield in 2019–2020 (Table 2). Thus, as shown in Fig 4, grain yield of KN199 increased significantly by 14.70%, 14.07%, and 9.16% at Gaocheng, Xinle, and Zhaoxian, respectively, under N180 compared to the N0’treatment in 2018–2019 growing season. Similarly, in 2018–2019, grain yield of JM585 increased significantly by 16.99%, 18.96%, and 9.72% at Gaocheng, Xinle, and Zhaoxian, respectively, under N180 compared to the N0’treatment. Furthermore, in 2019–2020, the grain yield variation observed for the two winter wheat varieties evaluated was consistent with that observed in the 2018–2019 season. On the other hand, in 2019–2020 at Gaocheng, the grain yield of KN199 and JM585 under the N0–N300 treatments significantly lower than 2018–2019. No significant differences in grain yield were detected among N180, N240, and N300 in either growing season.

**Fig 5:**
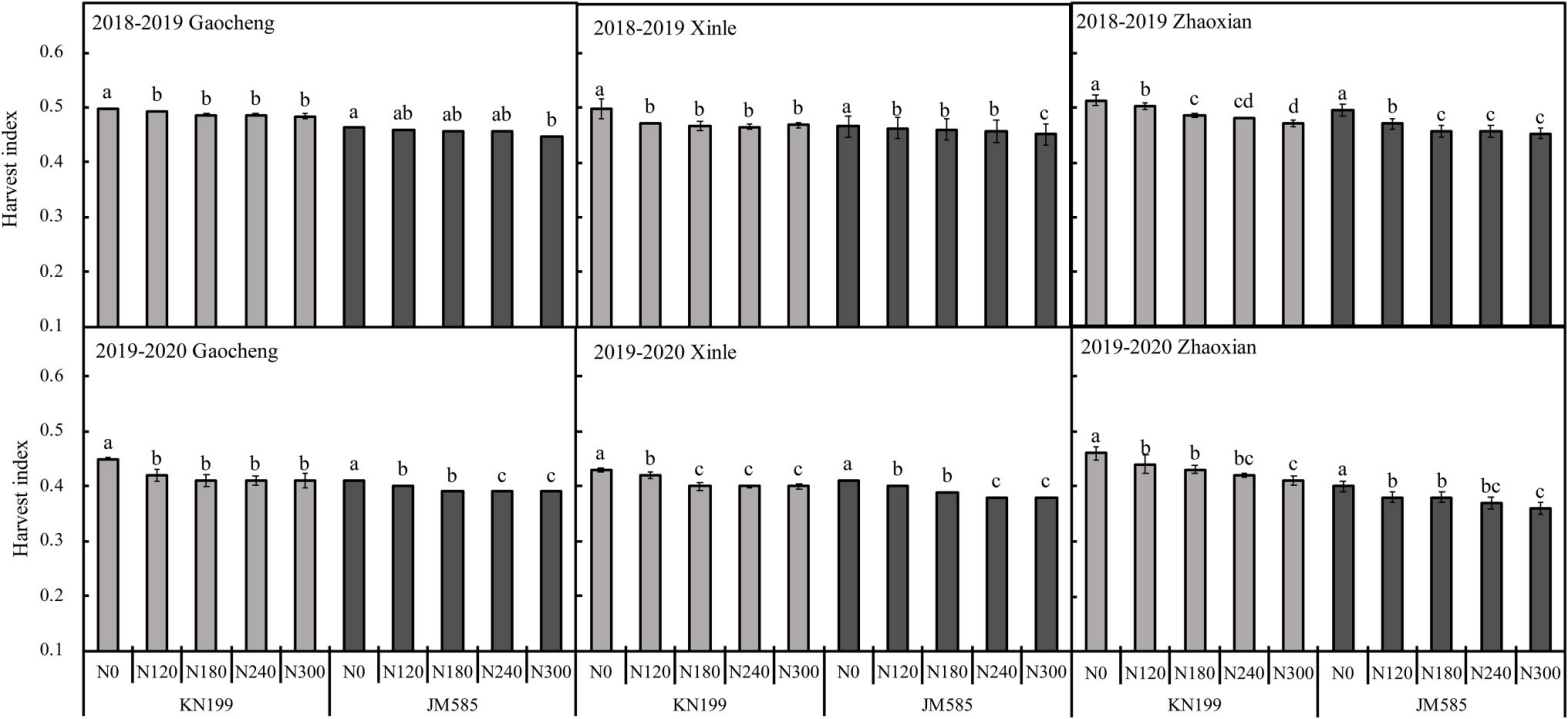
Harvest index of different winter wheat varieties under different nitrogen fertilization rates in three experimental sites during 2018–2020 seasons. Error bars represent one standard deviation from the mean. Letters above the bars are comparison results of leaf area index between different nitrogen treatments of the same variety. There is no significant difference between treatments with same letters.

Variety and N rate significantly affected HI in 2018–2020 while location significantly affected HI in 2018–2019. The interaction of L×V significantly affected the HI in 2018–2020, and L×N only affected the HI in 2019–2020 (Table 2). As shown in Fig 5, HI was highest under N0 and decreased as the N application rate increased. The range of HI values observed in 2018–2019 for KN199 under N0–N300 at Gaocheng, Xinle, and Zhaoxian, was 0.48–0.50, 0.47–0.50, and 0.47–0.51, respectively. Similarly, in 2018–2019, the range HI values observed that same year for JM585 HI was 0.45–0.46, 0.45–0.47, and 0.45–0.50, respectively. In 2019–2020, HI values for the two varieties of winter wheat were consistent with those observed in 2018–2019. HI values for KN199 were higher than those for JM585 across N application rates.

### Winter wheat nitrogen accumulation, N harvest index, and NUE

Location and N rate significantly affected total N accumulation in 2018–2020 while variety significantly affected total N accumulation in 2019–2020. The interaction of L×V significantly affected the total N accumulation in 2018–2020, and L×N, V×N only affected the total N accumulation in 2018–2019. Overall, location, variety, and N rate significantly affected grain N accumulation in 2018–2020. The interaction of L×V and L×N significantly affected the grain N accumulation in 2018–2020, and V×N only affected the total N accumulation in 2018–2019. Location, variety, and N rate significantly affected NHI in 2019–2020. The interaction of L×V and L×N significantly affected the NHI in 2018–2020. Location, variety and N rate in 2018–2020. The interaction of L×V, L×N and V×N significantly affected NUE in 2018–2020 (Table 2).

As shown in Table 3, total N accumulation in KN199 increased significantly by 28.09%, 28.00%, and 37.35% at Gaocheng, Xinle, and Zhaoxian, respectively, under N180 compared to the N0’treatment in 2018–2019 growing season. Similarly, in 2018–2019, total N accumulation in JM585 increased significantly by 40.16%, 34.83%, and 45.68% at Gaocheng, Xinle, and Zhaoxian, respectively, under N180 compared to the N0’treatment. Furthermore, when N input increased to N240, total N accumulation significantly increased in the three experimental locations. When N input increased to N300, the total N accumulation did not increase significantly. Grain N accumulation in KN199 increased significantly by 23.97%, 23.76%, and 28.41% at Gaocheng, Xinle, and Zhaoxian, respectively, under N180 compared to the N0’treatment in 2018–2019 growing season. Similarly, in 2018–2019, grain N accumulation in JM585 increased significantly by 40.16%, 34.83%, and 45.68% at Gaocheng, Xinle, and Zhaoxian, respectively, under N180 compared to the N0’treatment. NHI for KN199 was 0.86–0.83, 0.89–0.85, and 0.89–0.81 at Gaocheng, Xinle, and Zhaoxian, respectively, while NHI for JM585 was 0.85–0.81, 0.86–0.83, and 0.86–0.79 at Gaocheng, Xinle, and Zhaoxian, respectively. Furthermore, in 2019–2020, total N accumulation, grain N accumulation, and NHI and NUE variation for the two winter wheat varieties tested were consistent with the corresponding values obtained in the previous season. Total N accumulation and grain N accumulation increased with increasing fertilization rates, whereas NHI and NUE decreased with increasing fertilization rates.

**Table 3:** Nitrogen accumulation at maturity of different winter wheat varieties under different nitrogen fertilization rates in three experimental sites during 2018–2020 seasons.

2018–2019							2019–2020			
Treatment			Total nitrogen accumulation (kg ha^-1^)	Grain nitrogen accumulation (kg ha^-1^)	N harvest index	NUE	Total nitrogen accumulation (kg ha^-1^)	Grain nitrogen accumulation (kg ha^-1^)	N harvest index	NUE
Location	Variety	N rate								
Gaocheng	KN199	N0	208.17d	178.19d	0.86a	34.36a	201.26d	171.25c	0.85a	33.18a
		N120	231.40c	195.04c	0.84b	33.00b	242.03c	190.82b	0.79b	29.81b
		N180	266.65b	220.90b	0.83c	32.91b	258.91b	198.13b	0.77c	28.91bc
		N240	292.54a	243.09a	0.83c	30.44c	283.22a	213.29a	0.75d	28.14c
		N300	296.58a	244.71a	0.83c	30.40c	291.60a	217.45a	0.75d	28.11c
	JM585	N0	201.29d	170.64c	0.85a	34.41a	181.94d	148.78d	0.82a	32.66a
		N120	247.49c	204.98b	0.83b	31.95b	222.93c	174.15c	0.78b	30.35b
		N180	282.13b	232.52a	0.82b	30.24c	258.54b	192.22b	0.74c	28.26c
		N240	295.50a	240.03a	0.81c	29.57c	281.94a	207.79a	0.74c	27.69c
		N300	300.91a	243.06a	0.81c	29.20c	287.73a	210.27a	0.73c	27.48c
Xinle	KN199	N0	143.98d	128.34d	0.89a	38.07a	148.78d	124.87d	0.84a	35.87a
		N120	169.38c	148.08c	0.87b	35.04b	186.70c	154.67c	0.83b	32.17b
		N180	184.30b	158.84b	0.86c	35.05b	208.74b	167.43b	0.80c	32.61b
		N240	203.01a	173.65a	0.86c	32.81c	245.31a	195.19a	0.80c	28.84c
		N300	208.88a	178.14a	0.85c	32.35c	249.41a	196.26a	0.79d	28.46c
	JM585	N0	134.23d	116.06d	0.86a	38.26a	138.10d	113.82d	0.82a	34.85a
		N120	169.12c	145.17c	0.86a	35.54b	183.43c	146.65c	0.80b	32.37b
		N180	180.98b	154.68b	0.85b	35.40b	209.17b	163.54b	0.78c	30.56c
		N240	197.95a	167.60a	0.85b	33.02c	223.92a	170.77a	0.76d	28.43d
		N300	201.67a	170.05a	0.84c	32.03c	226.02a	171.64a	0.76d	28.52d
Zhaoxian	KN199	N0	164.44d	145.98c	0.89a	39.89a	167.47d	142.30c	0.85a	41.99a
		N120	184.18c	160.56b	0.87b	38.87b	205.01c	172.88b	0.84a	35.27b
		N180	225.87b	187.46a	0.83c	32.38c	259.01b	208.55a	0.81b	32.39c
		N240	241.76ab	196.98a	0.81d	31.35d	276.01a	215.54a	0.78c	30.98c
		N300	243.60a	198.11a	0.81d	30.90d	279.44a	216.17a	0.77c	30.75c
	JM585	N0	152.77d	131.23c	0.86a	42.29a	155.75d	126.84c	0.81a	37.78a
		N120	200.17c	167.37b	0.84b	34.26b	196.83c	154.54b	0.78b	32.47b
		N180	222.56b	178.18a	0.80c	32.21c	246.35b	184.17a	0.75c	29.01c
		N240	228.46ab	181.36a	0.79d	32.13c	258.87ab	188.80a	0.73d	28.02cd
		N300	237.42a	187.44a	0.79d	31.24c	269.72a	194.66a	0.72d	27.22d

Note: Different letters following data of same trait and variety indicate significant differences between nitrogen treatments (*P* < 0.05).

### Economic return

As shown in Table 4, In 2018–2020, water cost and cost of cultivation for two winter wheat varieties under five N treatment being same at three experiment location. Fertilizer cost increased as fertilizer rate increased in both winter wheat varieties. In 2018–2020, fertilizer cost for two winter wheat varieties under the N180 treatment being higher by 13%, 50% than N0, N120, being lower by 10%, 22% than N240, N300 treatment at three experiment location. Gross profit obtained from the two tested winter wheat varieties increased with N application rate across locations and over the two growing seasons. However, no significant further increases in gross profit were observed when N input exceed to 180 kg ha^-1^. Net return first increased and then decreased as fertilizer rate increased in both winter wheat varieties. In 2018–2019, net return obtained from KN199 was significantly the highest under the N180 treatment being higher by 1.49%–13.76%, 1.20%–16.21% than other treatment at Gaocheng and Xinle, under the N120 treatment being higher by 0.53%–6.14% than other treatment at Zhaoxian. Net return obtained from JM585 was significantly the highest under the N180 treatment being higher by 3.41%–19.25%, 3.36%–27.14%, 0.56%–6.85% than other treatment at Gaocheng, Xinle and Zhaoxian. As for 2019–2020, net return obtained from KN199 was significantly the highest under the N180 treatment being higher by 3.49%–20.48%, 3.79%–77.32%, 1.84%–23.13% than other treatment at Gaocheng, Xinle and Zhaoxian. Net return obtained from JM585 was significantly the highest under the N180 treatment being higher by 4.33%–66.96%, 3.02%–68.38%, 1.25%–26.41% than other treatment at Gaocheng, Xinle and Zhaoxian.

**Table 4:** Economic return (CNY ha^-1^) of different winter wheat varieties under different nitrogen fertilization rates in three experimental sites during 2018–2020 seasons.

2018–2019								2019–2020				
Treatment			Water cost	Fertilizer cost	Cost of cultivation	Gross profit	Net return	Water cost	Fertilizer cost	Cost of cultivation	Gross profit	Net return
Location	Variety	N rate										
Gaocheng	KN199	N0	1060a	1725e	3600a	16778b	10393b	1060a	1725e	3600a	12889b	6504b
		N120	1060a	2297d	3600a	18519a	11562a	1060a	2297d	3600a	14333a	7376ab
		N180	1060a	2585c	3600a	19244a	11999a	1060a	2585c	3600a	15081a	7836a
		N240	1060a	2873b	3600a	19356a	11823a	1060a	2873b	3600a	15105a	7572ab
		N300	1060a	3159a	3600a	19174a	11355a	1060a	3159a	3600a	15096a	7277ab
	JM585	N0	1060a	1725e	3600a	16348c	9963c	1060a	1725e	3600a	11185c	4800c
		N120	1060a	2297d	3600a	17889b	10932b	1060a	2297d	3600a	13230b	6273bc
		N180	1060a	2585c	3600a	19126a	11881a	1060a	2585c	3600a	15259a	8014a
		N240	1060a	2873b	3600a	19022a	11489ab	1060a	2873b	3600a	15214a	7681ab
		N300	1060a	3159a	3600a	19154a	11335ab	1060a	3159a	3600a	15207a	7388ab
Xinle	KN199	N0	1060a	1725e	3600a	11319b	4934a	1060a	1725e	3600a	10257c	3872b
		N120	1060a	2297d	3600a	12533a	5576a	1060a	2297d	3600a	13029b	6072a
		N180	1060a	2585c	3600a	12911a	5666a	1060a	2585c	3600a	14111a	6866a
		N240	1060a	2873b	3600a	13267a	5734a	1060a	2873b	3600a	14148a	6615a
		N300	1060a	3159a	3600a	13148a	5329a	1060a	3159a	3600a	14096a	6277a
	JM585	N0	1060a	1725e	3600a	10667b	4282a	1060a	1725e	3600a	9968c	3583c
		N120	1060a	2297d	3600a	12067a	5110a	1060a	2297d	3600a	12111b	5154b
		N180	1060a	2585c	3600a	12689a	5444a	1060a	2585c	3600a	13278a	6033a
		N240	1060a	2873b	3600a	12800a	5267a	1060a	2873b	3600a	13389a	5856ab
		N300	1060a	3159a	3600a	12884a	5065a	1060a	3159a	3600a	13252a	5433ab
Zhaoxian	KN199	N0	1060a	1725e	3600a	14067b	7682a	1060a	1725e	3600a	13074b	6689b
		N120	1060a	2297d	3600a	15111a	8154a	1060a	2297d	3600a	15044a	8087a
		N180	1060a	2585c	3600a	15356a	8111a	1060a	2585c	3600a	15481a	8236a
		N240	1060a	2873b	3600a	15452a	7919a	1060a	2873b	3600a	15513a	7980a
		N300	1060a	3159a	3600a	15450a	7631a	1060a	3159a	3600a	15496a	7677a
	JM585	N0	1060a	1725e	3600a	13941b	7556a	1060a	1725e	3600a	10630c	4245b
		N120	1060a	2297d	3600a	14963a	8006a	1060a	2297d	3600a	11963b	5006ab
		N180	1060a	2585c	3600a	15296a	8051a	1060a	2585c	3600a	12611ab	5366a
		N240	1060a	2873b	3600a	15430a	7897a	1060a	2873b	3600a	12833a	5300a
		N300	1060a	3159a	3600a	15354a	7535a	1060a	3159a	3600a	12666ab	4847ab

Note: Different letters following data of same trait and variety indicate significant differences between nitrogen treatments (*P* < 0.05).

### Optimization of variety and N application rate based on yield and economic return

**Fig 6:**
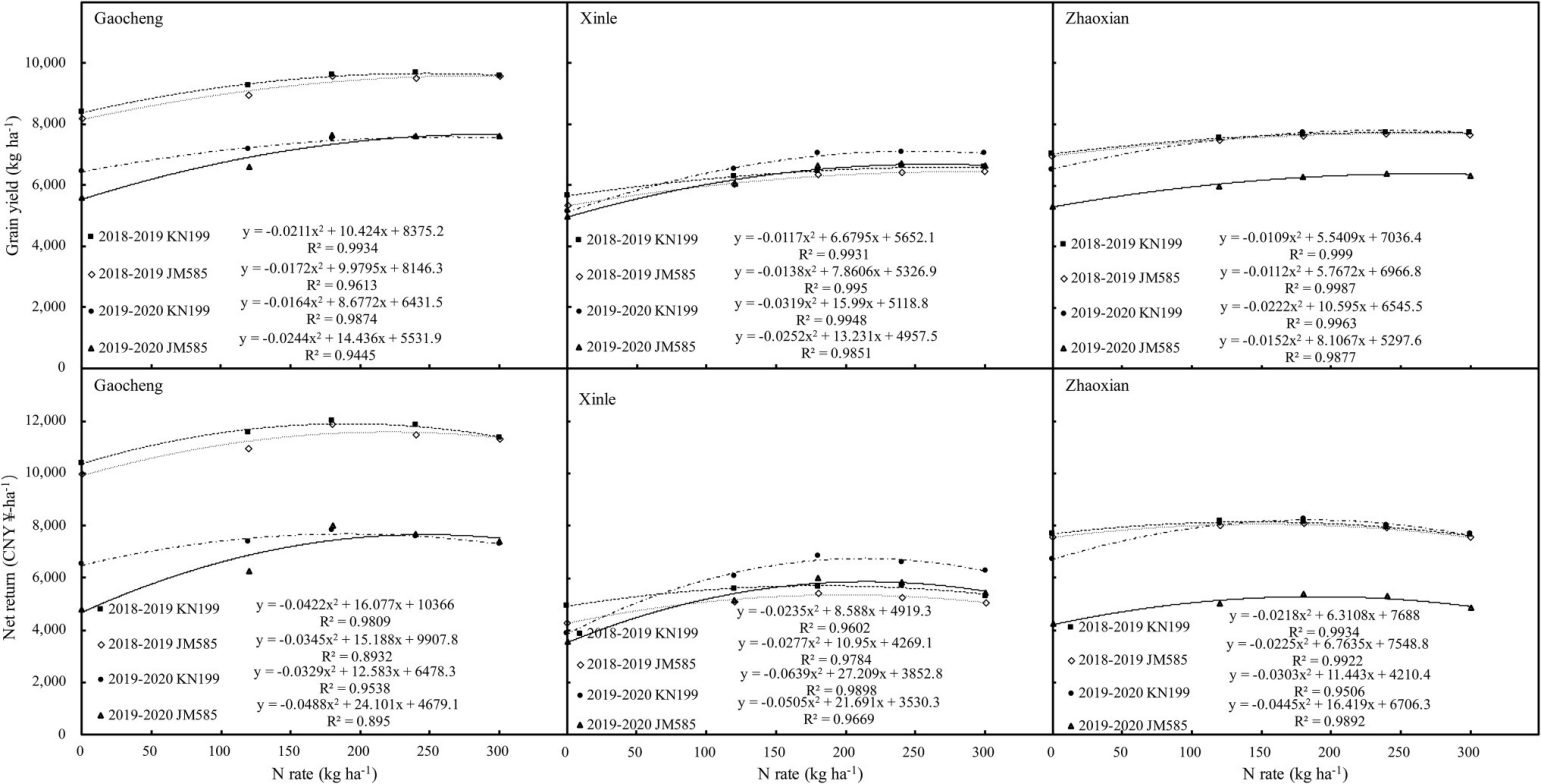
Correlation of winter wheat grain yield and economic return to nitrogen fertilization rates.

The data collected in this study over two growing seasons were comprehensively analyzed by considering the effects of different fertilization rates. N application rate was treated as an independent variable, and grain yield and net return were considered as response variables. The data were analyzed using the fertilizer effect function, and a unitary quadratic regression equation was determined, which was used to calculate the fertilizer amount required to maximize grain yield and net return (Fig 6). In 2018–2019, the maximum yield from KN199 was achieved with N application rates of 247.0, 285.5, and 254.2 N kg ha^-1^ at Gaocheng, Xinle, and Zhaoxian, respectively. In turn, net return was maximized with the application of 190.5, 192.2, and 144.7 N kg ha^-1^ at Gaocheng, Xinle and Zhaoxian, respectively. In the same period, the maximum yield from JM585 was achieved with N application rates of 290.1, 284.8, and 257.5 N kg ha^-1^ at Gaocheng, Xinle, and Zhaoxian, respectively, while net return in this case was maximized with the application of 220.1, 246.9, and 150.3 N kg ha^-1^ at Gaocheng, Xinle, and Zhaoxian, respectively. In turn, in the 2019–2020 growing season, the maximum yield from KN199 was achieved with N application rates of 264.6, 250.6, and 238.6 N kg ha^-1^ at Gaocheng, Xinle, and Zhaoxian, respectively, while net return was maximized with the application of 191.2, 212.9, and 184.5 N kg ha^-1^ at Gaocheng, Xinle, and Zhaoxian, respectively. Meanwhile, in the same period, the maximum yield from JM585 was achieved with N application rates of 295.8, 262.5, and 266.7 N kg ha^-1^ at Gaocheng, Xinle, and Zhaoxian, respectively, while net return was maximized with the application of 223.3, 214.8, and 188.8 N kg ha^-1^ at Gaocheng, Xinle, and Zhaoxian, respectively.

When the N application rate exceeded 180 kg ha^-1^, grain yield did not increase significantly. Furthermore, at rates lower than the optimal N application rate, the net return of winter wheat increased with the application rate; however, when higher than the optimal rate, the return gradually decreased. Altogether, data indicated that the optimal N application rate in the Piedmont plain of the Taihang Mountains in China, ranged from 144.7 to212.9 kg ha^-1^ for KN199 and from 150.3 to 247.0 kg ha^-1^ for JM585.

## Discussion

### Effects of nitrogen application rate on LAI, aboveground biomass, grain yield, and harvest index of winter wheat

Variety, amount of irrigation, and fertilizer available for uptake directly affect the growth and development of wheat, thereby affecting final grain yield [26,27]. Suitable irrigation amount, N application rate, and variety can significantly increase LAI, aboveground biomass, and grain yield of winter wheat, while N uptake increases with increasing irrigation and N fertilization [28,29]. Previous studies found that LAI, biomass production, grain yield, and yield are positively affected by N fertilization, but negatively affected by water stress [30,31]. In the experiments reported herein, N application rate affected grain yield and LAI of the genotypes under evaluation; furthermore, genotypic responses varied with environment, i.e., with site and year [32]. LAI and aboveground biomass were significantly higher under the N180 treatment than under the N0’treatment across locations. When N input increased to N240, there were no significant differences in KN199, and when increased to N300, there were no significant differences in JM585 with respect to LAI or aboveground biomass. When the N application rate exceeded 180 kg ha^-1^, grain yield did not increase significantly; as what increased mostly was the dry matter accumulated in vegetative organs, while HI decreased concomitantly. Guttieri et al. [33] studied the effect of N fertilization on the grain yield of different genotypes. Yue et al. [34] found that wheat grain yield increased with an increase in N supply but excess N did not increase grain yield nor grain N accumulation in five experimental sites. Consistently, Zhang et al. [35] showed that 190 kg N ha^-1^ can be applied in Beijing, China, to maintain a steady yield for at least two years. In this study, grain yield increased with increasing N application rate but when it exceeded 180 kg ha^-1^, the yield of the two varieties tested did not increase significantly. In 2019–2020, at the Gaocheng experimental location, the grain yield for KN199 and JM585 decreased by 21.27–23.18% and 20.02–26.69%, respectively, compared with the grain yield recorded in the 2018–2019 growing season. This was attributed to a reduction in the number of grains per panicle owing to freezing damage at the panicle differentiation stage and continuous rain during the flowering period (Fig 1).

### Effects of application rate on N accumulation and NUE

Excess N application results in reduced NUE and soil pollution [36]. Improving NUE facilitates the rational utilization of agricultural resources [37]. Zheng et al. [18] concluded that N accumulation in winter wheat increases with increasing N application rate; however, grain N accumulation decreases at N application rates above 240 kg N ha^-1^. In this study, total N accumulation and grain N accumulation increased with increasing N application rate and when the latter exceeded 240 kg ha^-1^, they remained increased in a non-significant way. Presumably because of differences in planting area and wheat variety. In addition, the results of this study showed that the N240 and N300 treatments increased total plant N uptake; however, grain N uptake and grain yield did not increase. Indeed, our results showed that maximum NHI was obtained under the N0’treatment in 2019–2020 at the three experimental sites; yet, conversely, the accompanying yields were the lowest. This may be due to frost damage during the panicle differentiation period and continuous rain during the flowering period of 2019–2020 (Fig 1), the number of grains per spike was reduced, which resulted in a decrease in grain yield. Several studies have reported a NUE below 30% for winter wheat [18,38]. Studies have shown that wheat varieties differ for NUE; high-N varieties (such as varieties with high uptake efficiency and utilization efficiency) also have high plant dry matter yields and grain yields under conditions of insufficient N supply [39]. In this study, NUE generally decreased and differed only slightly with increasing N application rate across sites in both varieties; furthermore, it decreased in both with an increasing N application rate. NUE of KN199 and JM585 ranged within 30.4–39.89 and 28.11–41.99, and 29.2–42.29 and 27.22–37.78 in the 2018–2019 and 2019–2020 growing seasons, respectively. As it can be seen, NUE was higher in the first than in the second growing season, mainly owing to the high rainfall that caused the accumulation of dry matter in plant vegetative organs in the latter. Our results demonstrated that excessive N fertilization reduced grain N uptake, grain yield, and NUE. Therefore, environment-dependent site-specific genotype and N application rate recommendations should be promoted, in accordance to prevailing environmental conditions or seasonal expectations.

### Combined effects of variety and nitrogen application rate

Many researchers have elucidated the relationships among amount of irrigation, fertilizer input, and crop yield using a combination of multivariate regression and spatial analyses [40,41]. The optimal N application rate for wheat varies with soil conditions in the planting area. Previous studies on wheat have shown that an irrigation of 240 mm and an N application rate of 150–210 kg N ha^-1^ can maintain higher productivity in the entire system with higher resource use efficiency and lower N_2_O emissions in the NCP [11]. In this study, the relationships between fertilizer cost and crop yield were further analyzed and the net return for the maximum values of these parameters was assessed at three experimental sites. The study data suggest that the optimal N fertilizer rates to use at the Piedmont plain of the Taihang Mountains for the sustainable production of winter wheat varieties KN199 and JM585 are 144.7–212.9 and 150.3–247.0 kg ha^-1^, respectively.

## Conclusions

Under limited irrigation conditions, LAI, dry matter accumulation, grain yield, and nitrogen accumulation of the two evaluated wheat varieties in the study sites increased with increasing N application rate; however, HI, NHI, and NUE decreased. The optimal fertilizer amounts for winter wheat varieties KN199 and JM585 varied within the ranges of 144.7–212.9 and 150.3–247.0 kg ha^-1^, respectively, as these conferred the highest economic return, while facilitating water and fertilizer conservation, decreasing groundwater pollution risk, and maintaining high grain yield. The results of this study are of great significance for the scientific management of winter wheat fertilization in the Piedmont plains of the Taihang Mountains of China, which may be similar to many other arid and semi-arid winter wheat production areas around the world.

## Supporting information

S1 Data (XLSX)
