# Supplementary material for: Optimizing nitrogen fertilizer amount for best performance and highest economic return of winter wheat under limited irrigation conditions
Source: PLoS One. 2021 Nov 29;16(11):e0260379. doi: 10.1371/journal.pone.0260379 (PMC8629221; doi:10.1371/journal.pone.0260379)
Supplement: Attachment — Submitted filename: Response to Reviewers.docx [file pone.0260379.s003.docx]

**Detailed Response to Reviewer and Editor**

**(****PONE-D-21-25804)**

Dear Editor,

We appreciate the opportunity to revise the manuscript (Manuscript ID PONE-D-21-25804). We would like to express our sincere thanks to you and the reviewers for your constructive and valuable comments, which are very helpful for improving the manuscript. We have revised the text as described below and also provide point-by-point response to each comment. Changes in the revised manuscript are indicated in yellow font. Hope this revised version of the manuscript would be qualified for publication in PLOS ONE.

Reviewer #1

We very much appreciate your time and kind detailed comments on the manuscript. Revises have been done accordingly.

1. Page 9, lines 134–136: Kindly adopt the following manner when stating the methods of fertilization and irrigation: “N fertilizer was spread… a water meter was connected… then the water pipe was connected to the plots…”

**Response:** Revised as advised.

2. Page 10, line 144: Kindly adjust as follow: “pesticides”.

**Response:** Revised as advised.

3. Page 13, lines 197–199: Kindly adjust the sentence as follow: “Moreover, in 2019-2020, at Xinle and Zhaoxian experimental locations, the LAI of JM585 increased significantly by 4.34% and 11.95%, respectively when the N input increased from N180 to N240”.

**Response:** Revised as advised.

4. Page 15, lines 213–214: Kindly adjust the sentence as follow: “aboveground biomass significantly increased in Xinle and Zhaoxian experimental locations.”

**Response:** Revised as advised.

5. Page 18, lines 259–260: Kindly adjust the sentence as follow: “total N accumulation significantly increased in the three experimental locations”.

**Response:** Revised as advised.

6. Page 20–21, lines 284–292: Kindly compare statistically your results in this section. In other words, compare your results in the following manner: “Net return was significantly the highest (you state the growing season, location and variety) being higher by … (you state the range between the minimum net return and the maximal one). In this manner, you give your valuable study and its results their right and you avoid reading the table. I’m glad seeing the ANOVA test added for gross profit and economic return as recommended and your results look now more valuable. Good work !!

However, kindly add the ANOVA test also for water cost and cost cultivation (all “a” as being the same). Kindly add also the ANOVA test for fertilizer cost in order to detect if there is significance between all N fertilization rates or not, and discuss the obtained results.

**Response:** Revised as advised.

7. Page 26, lines 367–368: Kindly adjust the sentence as follow: “and when the latter exceeded 240 kg ha^-1^, they remained increased in a non-significant way”.

**Response:** Revised as advised.

8. Page 26, line 373: Kindly adjust the sentence as follow: “This may be due to frost damage…”

**Response:** Revised as advised.

**In addition,**

1. In economic analysis, “benefit” was replaced with “return” and the changes were marked in yellow font.

2. The manuscript format was modified to meet the requirements of PLOS ONE. We added figure captions appear directly after the paragraph in which they are first cited in the manuscript.

3. Table and figure captions were revised.
